# Postoperative neurogenic pulmonary edema in pediatric neurosurgery – clinical presentation and management in 2 cases: A case report

**DOI:** 10.1097/MD.0000000000044865

**Published:** 2025-10-03

**Authors:** Yifan Feng, Yue Zhang, Dingyi Yu, Yun Li

**Affiliations:** a Department of Anaesthesiology, Beijing Tiantan Hospital, Capital Medical University, Beijing, China.

**Keywords:** case report, intracranial hypertension, neurogenic pulmonary edema, pediatric neurosurgery, postoperative complications

## Abstract

**Rationale:**

Neurogenic pulmonary edema (NPE) is a rare but potentially life-threatening complication of severe central nervous system injury. Postoperative NPE is particularly uncommon in pediatric patients, and its pathophysiological mechanisms remain incompletely understood. Our objective is to review the perioperative management of such patients and provide recommendations and insights.

**Patient concerns:**

We report 2 pediatric cases of NPE following neurosurgical procedures. The first case involved a 2-year-old girl who developed acute respiratory distress immediately after undergoing a suboccipital craniotomy. The second case involved a 6-year-old girl who presented with progressive respiratory failure 1 day after an endoscopic third ventriculostomy.

**Diagnoses:**

Both patients exhibited clinical and radiographic findings consistent with NPE, including acute-onset pulmonary infiltrates and hypoxemia in the absence of primary cardiac dysfunction. The diagnosis was established based on characteristic postoperative timing, imaging studies, and the exclusion of other causes of pulmonary edema.

**Interventions:**

Both patients received timely mechanical ventilation and diuretic therapy. Supportive care, including oxygen supplementation and hemodynamic optimization, facilitated recovery.

**Outcomes:**

Both cases had favorable clinical outcomes, with resolution of respiratory symptoms and no long-term sequelae.

**Lessons:**

The differing onset and presentations of NPE in these cases underscore potential variations in intracranial pressure dynamics and surgical factors. Early recognition, prompt diagnosis, and immediate intervention are essential for optimal patient outcomes. Pediatric neurosurgical patients require vigilant postoperative monitoring for respiratory complications, particularly in the context of intracranial pressure changes. Strategic intraoperative management and supportive care play a crucial role in improving prognosis.

## 1. Introduction

Neurogenic pulmonary edema (NPE) is a rare but serious complication characterized by acute respiratory distress and pulmonary edema following central nervous system (CNS) injury.^[1,2]^ The pathophysiology remains incompletely understood, but current theories suggest that acute intracranial hypertension and sympathetic overactivation lead to elevated pulmonary vascular pressure and increased capillary permeability. These processes culminate in fluid leakage and pulmonary edema.^[2–5]^

Although NPE is well-documented in adults,^[6–8]^ pediatric cases are rarely reported. Immature CNS development and limited compensatory mechanisms may increase susceptibility in children, particularly during neurosurgical procedures or in conditions involving intracranial hypertension. The rarity of NPE in this population complicates its timely diagnosis and treatment, underscoring the importance of recognizing its early signs. This report discusses 2 pediatric NPE cases following neurosurgical interventions with the aim of highlighting the clinical manifestations, management strategies, and implications for future practice.

## 2. Case presentation

### 2.1. Patient 1

A previously healthy 2-year-old girl weighing 12 kg presented with unexplained difficulty in walking, as reported by her family. magnetic resonance imaging revealed an intracranial space-occupying lesion accompanied by obstructive hydrocephalus. For further treatment, she was admitted to Beijing Tiantan Hospital, where a diagnosis of a posterior fossa tumor with supratentorial hydrocephalus was confirmed. Suboccipital craniotomy was performed under general anesthesia.

Preoperative evaluation revealed normal laboratory results, good airway condition, and stable baseline vital signs (blood pressure, 112/69 mm Hg; oxygen saturation, 99%; heart rate, 103 bpm). The girl was required to fast from food and water for 6 hours prior to surgery. Intubation was successfully performed after induction with sufentanil, rocuronium, and propofol. Anaesthesia was maintained using sevoflurane and remifentanil. The patient’s vital signs remained stable intraoperatively. Fluid management included 600 mL of crystalloid, 50 mL of colloid, and 130 mL of red blood cells, with 200 mL of urine output and 200 mL of blood loss. The surgery lasted 6 hours.

**Figure 1.**
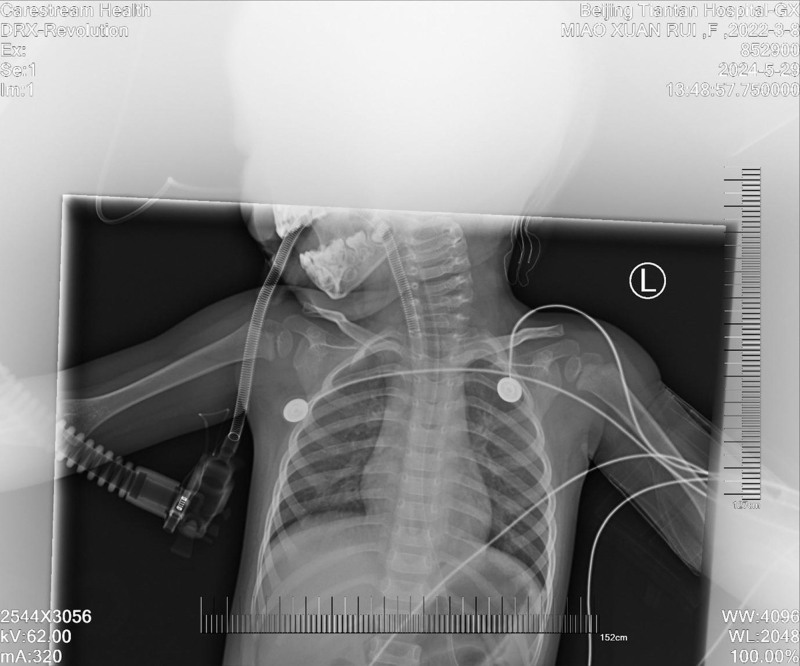
Postoperative chest X-ray image showing resolution of pulmonary edema in a 2-year-old patient after suboccipital craniotomy. X-ray imaging revealed the absence of pleural effusion and clear pulmonary fields, demonstrating successful treatment of acute NPE. NPE = neurogenic pulmonary edema.

Postoperatively, extubation failed due to hypoxemia (oxygen saturation < 90%) without signs of airway obstruction or wheezing. Chest auscultation revealed prominent bilateral wet rales and pink frothy sputum within the airway, strongly suggesting acute pulmonary edema. Furosemide (2 mg) and dexamethasone (5 mg) were administered, along with 100% oxygen and positive end-expiratory pressure via mechanical ventilation. In addition, the patient received intermittent intravenous sufentanil (1 µg) for sedation. The patient remained unconscious. After 1 hour, the wet rales were partially resolved and oxygen saturation improved to 95%. She was transferred to the intensive care unit (ICU) for continued ventilatory support. Over the following 48 hours, her condition improved significantly, with radiological resolution of pulmonary edema and successful extubation (Fig. 1). The patient experienced intermittent postoperative fever and was treated with antibiotics. She was discharged in stable condition 2 weeks later.

### 2.2. Patient 2

A previously healthy 6-year-old girl was admitted with a 1-month history of progressive head enlargement, without other symptoms. Imaging studies revealed obstructive hydrocephalus. She had previously undergone a ventriculoperitoneal shunt at a local hospital, which failed to relieve her symptoms. She was transferred to Beijing Tiantan Hospital for further evaluation and was scheduled to undergo endoscopic third ventriculostomy (ETV) under general anesthesia.

Preoperative vital signs were stable (blood pressure, 108/64 mm Hg; heart rate, 109 bpm; oxygen saturation, 100%), with normal airway assessment. She fasted for food and water for 6 hours preoperatively. The anesthesia induction protocol was the same as in Case 1, and intubation was successfully performed. Anaesthesia was maintained with remifentanil and desflurane. Intraoperative fluid input included 350 mL of crystalloid, with 200 mL of urine output and 10 mL of estimated blood loss. The surgery was uneventful, and extubation was successfully performed.

**Figure 2.**
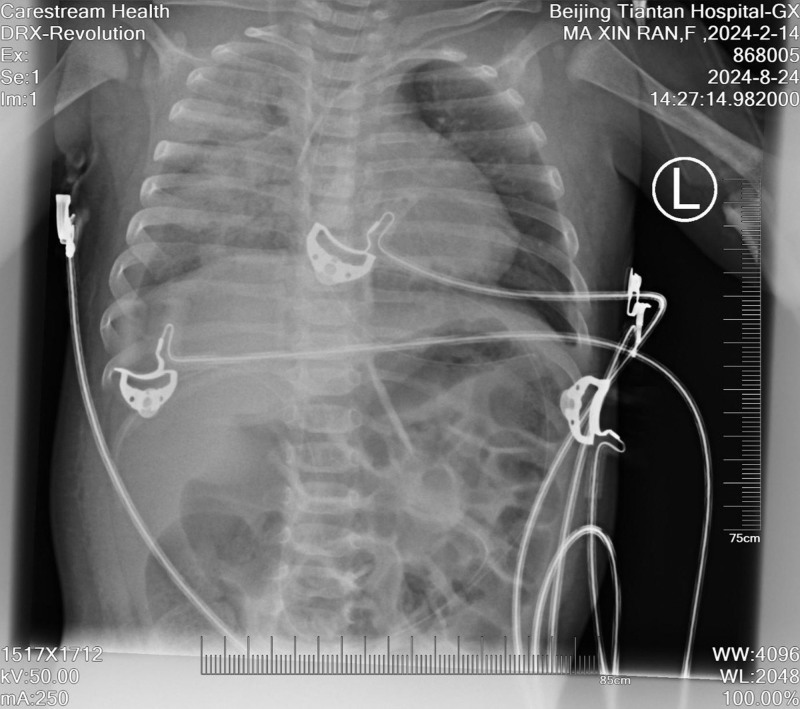
Postoperative chest X-ray image indicating pleural effusion and pulmonary edema in a 6-year-old patient after endoscopic third ventriculostomy. The image shows coarse lung markings and fluid accumulation.

On postoperative day 1 in the early morning, the patient suddenly developed hypoxemia (SpO₂ 80%–89%), tachycardia (heart rate 160–190 bpm), and mild cyanosis. Pink frothy sputum was observed in the airway, and chest auscultation revealed bilateral wet rales, indicating pulmonary edema. Endotracheal intubation and mechanical ventilation were promptly initiated. Diuretics were administered, and dexmedetomidine and remifentanil were used for sedation and analgesia. Chest radiography showed increased pulmonary markings and a small right-sided pleural effusion (Fig. 2). Cardiac biomarkers including B-type natriuretic peptide (BNP) and cardiac troponin I were elevated, suggesting myocardial stress. However, echocardiography showed no structural heart abnormalities, ruling out cardiogenic pulmonary edema and supporting a diagnosis of NPE. Oxygenation improved on the second postoperative day, and the patient was successfully extubated. She recovered well and was discharged 2 weeks later.

## 3. Discussion

NPE is a rare but potentially life-threatening complication that can arise after CNS injury or surgical intervention. Acute NPE typically develops within hours of insult, whereas delayed NPE may occur within 12-72 hours.^[2,9]^ Although the pathophysiology of NPE is not fully understood, leading hypotheses include overactivation of the sympathetic nervous system, intracranial hypertension, and dysregulated cardiac function. These mechanisms result in elevated pulmonary vascular pressure, capillary leakage, and fluid accumulation in the lungs.^[10,11]^

Few reports have described NPE in children.^[12,13]^ Similar to adults, NPE can be caused by a variety of factors, including subarachnoid hemorrhage,^[14]^ traumatic brain injury,^[15]^ intracranial hemorrhage,^[16]^ and epileptic status.^[17,18]^ In adult patients, NPE commonly occurs in emergency departments, neurosurgical units, or ICUs; however, its true incidence is difficult to estimate due to the lack of a standardized diagnostic definition. Furthermore, the incidence varies depending on the underlying etiology. Among patients with intracerebral hemorrhage, the incidence of NPE appears to be relatively high. Some studies report that up to 2-thirds of such patients develop pulmonary infiltrates during hospitalization, suggesting that NPE may be one of the most common underlying causes.^[19]^ In cases of acute brain injury, the estimated incidence of NPE ranges from 20% to 30%, often associated with poor outcomes. In pediatric patients, the immaturity of the CNS and limited autonomic regulatory capacity may render them more susceptible to NPE under these provoking factors.^[20]^ Currently, most information on pediatric NPE is derived from case reports, and the true incidence remains unclear. A retrospective review at our institution of 183 pediatric patients who underwent similar neurosurgical procedures showed that approximately 10% were transferred to the ICU due to pulmonary complications. However, due to atypical clinical presentations, rapid symptom resolution, and insufficient clinical awareness, many cases of NPE may go undiagnosed.

In this report, both pediatric patients developed NPE following neurosurgical procedures. In the first case, the onset was immediate after surgery. The operation was located in the posterior fossa, with manipulation close to the brainstem, which may have stimulated autonomic centers and triggered excessive sympathetic activation, resulting in acute postoperative pulmonary edema. Rapid fluctuations in intracranial pressure during surgery may also have led to hemodynamic instability, increased pulmonary capillary permeability, and aggravated the process. Previous studies have suggested that this mechanism involves strong activation of autonomic regulatory centers such as the solitary tract nucleus and the medulla during episodes of elevated intracranial pressure, triggering a “catecholamine storm”-like response that disrupts pulmonary circulation and promotes fluid extravasation.^[5]^ This mechanism has been supported by both animal models and clinical cases, particularly in conditions involving brainstem injury or pontine hemorrhage. Moreover, the patient’s failure to regain consciousness postoperatively suggested significant CNS depression, which may have further exacerbated autonomic imbalance.

In the second patient, the onset of symptoms was delayed, presenting on postoperative day 1. The child had preexisting chronic obstructive hydrocephalus and underwent ETV during surgery. This procedure relieves intraventricular pressure and disrupts the previously stable balance between intracranial and systemic circulation. The postoperative redistribution of cerebrospinal fluid (CSF) pressure may have triggered a secondary sympathetic reflex. Both animal and clinical studies have demonstrated that a rapid reduction in intracranial pressure (ICP) can induce elevated pressure within the pulmonary vascular bed and lead to capillary leakage via reflex mechanisms.^[21]^ This phenomenon is particularly pronounced in pediatric patients, as their cranial sutures are not yet fully closed and the CSF regulatory system is immature, making them more susceptible to systemic stress responses and subsequent NPE. In this case, symptoms developed on the first postoperative day, which we speculate to be associated with the dynamic process of ICP readjustment. Additionally, the patient exhibited elevated levels of brain natriuretic peptidebrain natriuretic peptide and cardiac troponin I. According to previous literature, such findings are consistent with the phenomenon of neurogenic myocardial stunning observed during acute neurological insults.^[22]^ Moreover, echocardiography revealed no structural abnormalities, effectively ruling out cardiogenic pulmonary edema. Compared to the first case, this patient tolerated extubation better postoperatively, suggesting a more gradual onset and milder severity of NPE.

In both cases, the patients experienced abrupt intracranial pressure (ICP) shifts during high-risk periods. The first case involved a sensitive surgical site near the brainstem and the decompression of supratentorial obstruction following tumor resection. In the second case, reconstruction of the CSF flow through ETV led to pressure redistribution. These represent typical intraoperative or postoperative scenarios with a high risk of structural ICP disruption. Therefore, we recommend that in pediatric patients at risk of sudden ICP fluctuations, decompression should be performed gradually and carefully, particularly during dynamic intracranial pressure changes.

Intraoperative management should include precise fluid control, continuous airway monitoring, intracranial pressure surveillance, and timely communication with the surgical team to ensure smooth operative manipulation.^[23,24]^ In addition, close monitoring of cardiac biomarkers is advised to help differentiate neurogenic from cardiogenic pulmonary edema.

## Author contributions

**Conceptualization:** Yun Li.

**Data curation:** Dingyi Yu.

**Investigation:** Dingyi Yu.

**Supervision:** Yun Li.

**Writing – original draft:** Yifan Feng.

**Writing – review & editing:** Yue Zhang.
